# A life of research on everyday sleep(iness)

**DOI:** 10.1093/sleepadvances/zpae076

**Published:** 2024-11-09

**Authors:** Torbjörn Åkerstedt

**Affiliations:** Department of Clinical Neuroscience, Karolinska Institute, Stockholm, Sweden; Department of Psychology, Stockholm University, Stockholm, Sweden

**Keywords:** sleep quality, sleep duration, mortality, gender, age, shift work, stress, sleepiness

## Abstract

This is a personal review of a research life focused on sleep in everyday life. It finds that irregular work hours shorten sleep duration and increase sleepiness, both subjectively and objectively (polysomnography). Also, experimental lab studies demonstrate reduced sleep duration (and sleep stages N2 and REM) when sleep is moved into the daylight hours (and the circadian upswing). Stage N3% seems not affected, and homeostatic experiments suggest that awakenings should not occur until the need for N3% or total spectral power has been satisfied. Furthermore, sleepiness is associated with increased alpha activity and slow eye movements, although the best indicator of dangerous sleepiness is subjective ratings (linked to perceptions of heavy eye lids). Everyday stress has very modest negative effects on objective sleep quality. Sleep loss as well as excessive sleep durations are linked to mortality, but with modest risk, and mainly in older individuals. Finally, objective sleep poorly reflects subjective sleep quality, and women appear to report poorer sleep than men, while objective data show better sleep quality in women. The discrepancy is considerably greater in older age groups.

Honored by the invitation to contribute to the series of “living legends,” I immediately wondered what to bring up. There are no rules, other than one’s “impact” on sleep science? That could be a bit too self-important and would have to be treated with delicacy. I think I ended up with the idea to bring up what I think has been most fun to search for (whatever “fun” means), with a focus on sleep regulation in daily life, including sleepiness and other outcomes. After all, who would be interested in sleep if one does not get sleepy (or fatigued, error prone, unhappy, ill, etc.) from sleep loss? Bernie Webb once burst out (rather long ago) that “the only thing we know about the effects of sleep loss is that it makes us sleepy.” The other focus is nonclinical, *everyday* sleep regulation and sleepiness, a relatively minor niche in vast sea of clinical research.

Looking at the reasons for entering sleep research, I note that other papers in the living legends series often bring up serendipity. That goes for me too. However, one also notes the prevalence of mentors that offer the young researcher a place in a sleep lab and guides him/her through the first steps. This is also followed by moves to other labs and new mentors in a progression of scientific development and finally with skill and luck a move into a sleep lab of one’s own.

In comparison, I feel a bit of an orphan with respect to sleep research. Instead, I was recruited as a research assistant to Dr Paula Patkai, who was part of the research group of the legendary stress researcher Professor Marianne Frankenhaueser, who did exciting research on catecholamines and everyday (and laboratory) stress, as well as educate us in physiological psychology. Paula had received funding for research on shift work and stress (with a heavy emphasis on catecholamines), so this is where my research activities started. Together, I and my corecruits and friends, Kerstin Pettersson and Per Dahlgren, spent the nights and days collecting urine and performance measures from shift workers in industries of different types. We noticed, however, that both we and the shift workers became very sleepy and obtained too little sleep.

The next step was Karolinska Institute and the “Laboratory for Clinical Stress research” in the Department of Psychiatry, led by the father of Swedish Stress Research, Dr Lennart Levi and Dr Jan Fröberg, who both encouraged continued research into shift work and circadian rhythms. Their 72 h studies of shooting performance around the clock were quite heroic. I came in at the end of this series and of course noted the increase in sleepiness across time and the strong circadian influence. This inspired us (with Lars Torsvall as a new member of our small group) to a number of field studies of around-the-clock work and its effect on circadian patterns of hormones, electrolytes, performance, fatigue, and health perception. Again, we lost a lot of sleep and spent many a night napping in industrial locker rooms and deserted train stations. Sleep problems and sleepiness continued cropping up in questionnaires, and I realized that we were missing something. Hence, I drifted into sleep research.

The sleep research started as a collaboration with my friend Mats Gillberg at the Swedish Defence Research Institute (FOA). He also was interested in sleep. I started to spend a lot of time there, and Mats found facilities that could be turned into a make-shift sleep lab. Together, we educated each other in sleep research with the help of a leftover Grass machine, the scoring manual of Rechtschaffen and Kales, volunteering colleagues, and the rather modest research literature on sleep physiology. With the help of research assistants, we were almost like a real sleep lab (we thought). Our first study was on of 64 h of sleep deprivation with polysomnography (PSG) recorded before and after. We worked around the clock and collected urine for catecholamines and electrolytes (out of habit). We saw the performance effects, the sleepiness, the deep postdeprivation sleep, the loss of memory for forced awakenings during recovery sleep, and we were very pleased with ourselves [1]. As a bonus, we also managed to be the first to show the close circadian covariation of urinary melatonin and sleepiness [2].

The success of the first study increased our appetite, and we improved on our skills via meetings with a rather small group of researchers engaged in shift work and circadian rhythms research (the Night and Shift Work Society). We met for scientific exchange in rather secluded places, away from distractions, and with ample time for intense discussion throughout the evenings, fueled by the odd bottle of wine or beer. Much of the initiative came from Professor Joseph Rutenfranz at the German Institute of Work Physiology in Dortmund, in collaboration with Associate Professor Peter Knauth. The meetings included key names such as William Colquhoun, Mary Loban, John Mills, James Wedderburn, Odile Benoit, Franz Halberg, and younger researchers like Simon Folkard, Jim Waterhouse, Jean Foret, Jürgen Zulley, and many others. The inspiration from these regular meetings was enormous, and we felt we were becoming part of a research community. The opportunities to have unlimited access to friendly big names as well as young colleagues, resulted in a feeling of euphoria. Other important groups that organized informal meetings in this golden age was that in Zürich—Alexander Borbély and Irene Tobler, with inputs also from Serge Daan and Domien Beersma in Groningen. The group in Basel contributed with meetings in Tuscan villas or within the dark walls of old Swiss castles, organized by Anna Wirz Justice. Making dinner together, and sleeping in sofas and arm-chairs wherever they could be found, strongly counteracted ivory tower mentality. And, of course, of paramount importance were Jürgen Aschoff and Rütger Wever and others in Erling Andechs (Germany)—the home of the famous bunker studies that set the standard of much circadian research in humans. A friendly approval from Jürgen Aschoff brightened the day for a long time, but one had the feeling that he could look with some amusement at younger researchers. One day I and Simon Folkard was discussing research with him, and he asked us “how old are you, boys?.” We answered “36,” and he commented “you are a bit cocky for your age, aren’t you?”

We also benefitted a lot from a continued exchange of views with researchers and friends like Charles Czeisler, David Dinges, Mark Rosekind, and Jim Horne. They graciously received me for some mini-sabbaticals. I think that, unconsciously, they became role models, and I was a bit envious of their success.

Gradually we grew up; however, the Laboratory for Clinical Stress Research became a government financed “Institute for Psychosocial Environmental Medicine” (IPM) (housed within Karolinska Institute). This turned me into a professor with a permanent position (no more any personal monetary worries). We even got a real isolation unit sleep lab, and we were joined by Göran Kecklund, who became an important pillar in our research structure, and Jens Nilsson who became the manager of the sleep lab. In a final step, I became the director of IPM, which moved to Stockholm University, giving us good financial conditions (although I retained an adjunct and later part-time position at Karolinska). Finally, there was a move back to Karolinska. On the whole, I felt I have been working at the same place most of the time. Now that many of the cocky young researchers of the early years are not so young anymore, and many of us are approaching our expiration date; it seems an appropriate time to take stock.

## Shift Work and Objective Sleep

After our first attempt at PSG research, we still also continued our “traditional” shift work research. As brought up above, our early questionnaire studies (and those of others) had shown that shift workers complained of poor sleep, particularly after the night shift (see review) [3], as well as sleepiness late on the night shift, but intermediate sleepiness on the morning shift. Almost all evidence came from cross-sectional studies, but in two longitudinal natural experiments, we found that sleep problems, poor sleep, and poor health were reduced after leaving shift work (with night shifts), but increased after entering it [4, 5]. This strengthened our interest in shift work research, in particular in terms of objective sleep.

When we started, only little was known about objective/physiological sleep, probably because it was difficult to bring shift workers into the sleep lab for polysomnographic (PSG – a combination of EEG, EOG, and EMG) recordings. Therefore, with the advent of ambulatory recording equipment, developed by Helgi Kristbjarnarson (also a good friend in the Nordic sleep community) with his Embla machine, our first venture into the real world using polysomnographical recordings involved a study of train drivers sleeping at home after a day shift and after a night shift, inspired by the first study of this type [6]. This was our first experience of entering other peoples’ bedrooms—a bit unnerving at first. Our study showed 3 h shorter sleep for day sleep after a night shift, as well as less stage N2 and REM (both are linked to TST – total sleep time) [7]. Stage 3 + 4 (slow wave sleep (SWS) or today’s Stage N3) was not much affected. Similar observations were made in a study of more traditional 3-shift work in the paper industry [8]. The pronounced daytime sleep truncation after the night shift, despite the increased time awake, was very inspiring and was interpreted as circadian interference with sleep. Hints of this had come from the napping around-the-clock studies by Carskadon et al and from experiments by Webb and Agnew on temporal shifts in sleep [9, 10]. In both of our studies above, also premorning shift sleep duration was reduced, but for more trivial reasons, people simply had to cut sleep short in order to get to the early shift in time. Notably, Stages 3 and 4 were not really affected, although REM sleep and stage 2 were reduced in proportion to TST.

## Experiment of PSG Sleep and Time of Day

The conclusions on circadian interference with sleep in the shiftwork studies were only assumptions, since our own studies had participants sleeping in their homes with possibilities of interference from light and noise during the daytime. The study by Foret and Benoit also had the problem of most participants’ day sleep taking place in dormitories, which might add further interference [6].

Hence, a laboratory study was needed (with non-shift workers), and we set out to record sleep at different times of day, with bedtimes at 2300 h, 0300 h, 0700 h, 1100 h, 1500 h, 1900 h, and 2300 h after being awake for 1600 h before the first bedtime at 2300 h and adding 4 h per displacement [11]. Importantly, the time of rising was ad lib. The instruction was “rise when you have slept enough.” The participants came to us once a week after recovery. The traditional shift worker would likely start his post–night-shift sleep around 0700 h, but some used other strategies of displaced sleep, so we wanted to cover the entire spectrum of possible bedtimes.

**Figure 1.**
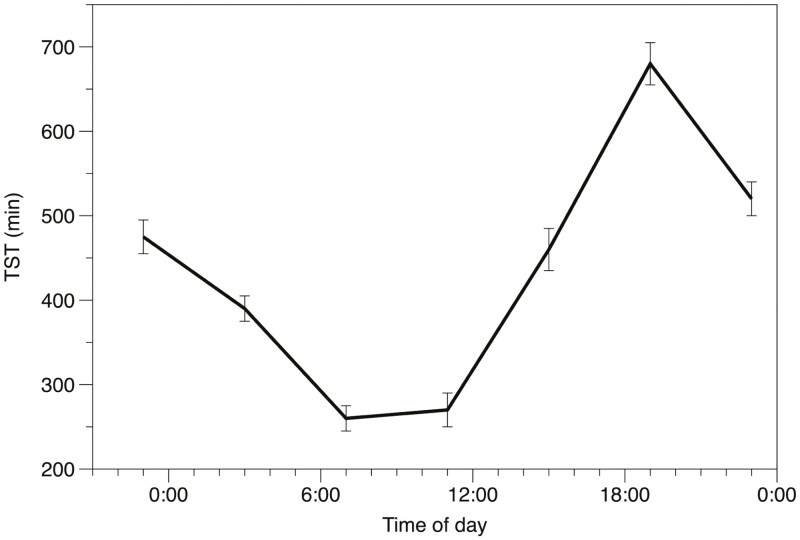
Sleep duration after bedtimes at different times of day and ad lib sleep duration. Mean ± se [11].

The results showed a pronounced U-shape (Figure 1) for TST and most PSG variables. The longest sleep occurred for the bedtime at 1900 h (> 3 h longer than after normal bedtime at 2300 h) after a normal time awake (16 h), and it was assumed that sleep pressure helped maintain sleep for some hours, with high metabolism around the circadian peak and then being maintained partly by the downward swing of the circadian system, followed by an awakening after the circadian system became activated toward the early morning. The sleep that started at 2300 h, after 40 h of time awake, was considerably shorter than that at the 1900 h bedtime after 36 h awake, and only marginally longer than that at 2300 h after 16 h awake. Thus, the effect of time awake on TST was modest. Interestingly, SWS did not show a circadian rhythm but remained at approximately the same level throughout. The latter made us suspect that the circadian regulation was not sufficient to interfere with SWS. We got the impression that sleep was not really “permitted” to terminate until a need for SWS had been satisfied.

It should be mentioned that the year before, Charles Czeisler’s group had shown in a seminal study of forced desynchronization under long time isolation that sleep duration was shortened when rectal temperature was close to its circadian maximum [12]. Not exactly the same as our time of day effect, but still reflecting the same circadian interference with sleep. Quite a few related studies were published by the same group across the subsequent years.

## Homeostatic Effects

The circadian influence on sleep duration was apparently considerable, but what about the homeostatic effect? Most experimental studies of sleep deprivation have had a set time in bed (TIB), which interfered with conclusions on homeostatic effects on sleep duration. The study on displaced sleep above suggested a very modest effect of 40 h awake [11].

However, a better time to study the homeostatic effects on sleep duration would perhaps be the daytime, when the circadian interference would be strong and might better reveal its battle with sleep homeostasis (in this case prior sleep loss). To find out, we scheduled day sleep to 1100 h and prior night sleep to either 8, 4, 2, or 0 h of night sleep duration (always ending at 0700 h) to manipulate sleep need [13]. This yielded a pronounced dose–response pattern: 120 min TST after 8 h of prior night sleep, 159 min after 4 h of prior night sleep, 209 min after 2 h of prior night sleep, and 274 min for 0 h of prior night sleep (p < .001 for the difference across conditions). The result was interpreted as a compromise between homeostatic need for sleep and circadian interference.

**Figure 2.**
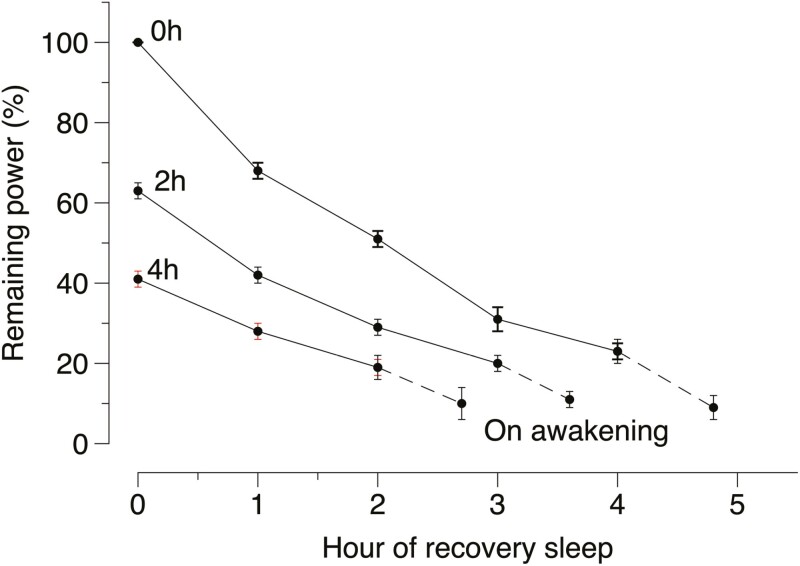
Total spectral power remaining to be recovered each hour during daytime sleep [13]. In percent of the total spectral power of night + daytime sleep for the 8 h sleep condition (mean ± se). The last point represents total spectral power remaining upon awakening (dashed line connects to last full hour of sleep).

The reason for the particular outcome was suggested by the response of individual sleep stages. Stage N2 and REM closely followed the effects on TST, showing a loss of recovery compared to night sleep. N3, however, was completely recovered during day sleep. This suggests that the need for N3 may have been satisfied before an awakening was “allowed” to occur. A similar result was obtained when total power (from spectral analysis 0.5-30Hz) was applied to the data [13]. Awakening occurred when almost all the total loss of delta power had been recovered (Figure 2). Hence, what we suggested that awakenings should not occur as long as there is a need for N3, but after that, the circadian influence determines sleep duration. Unfortunately, we did not have the resources to test several circadian phases for sleep with prior sleep restriction. To the best of my knowledge, such a study has not been carried out yet. The findings are reminiscent of the homeostatic recovery role of SWS proposed by Feinberg [14] and preceded by findings of Webb and Agnew [15].

## Circadian and Homeostatic Sleep Regulation and Modeling

As mentioned, Jürgen Aschoff and his group at Erling Andechs had pioneered the circadian/sleep interrelationship in studies that described the desynchronization of the circadian rhythm of temperature and sleep, as well as the influence of light [16, 17]. Sleep, however, was not really focused on, other than as a time-in-bed period, but started to appear in their work some years later [18]. In addition, as mentioned above, Charles Czeisler published his seminal work on sleep and circadian interaction [12]. However, also here, the details of sleep and its homeostatic character took some time to appear [19, 20].

One of the more entertaining scientific debates (or perhaps quarrels) concerned the existence of one or several oscillators (the driving force of the sleep/wake alternation) reviewed (by Borbély and Achermann) [21]. Gradually, it appeared that for human sleep/wake regulation, only one oscillator plus a homeostatic process were needed, and the seminal “two-process model of sleep regulation” (TPM) was presented by Borbély et al [22], with some input from our study of displaced sleep [11]. This model came to dominate the thinking of sleep regulation at that time and still does.

## Objective Sleepiness on the Job and in the Lab

While sleep is fascinating, it probably would not enjoy the present level of interest if recuperation after sleep loss would not be affected. For our part, our early studies of shift workers (with night shifts) in general showed a marked increase in self-rated sleepiness during the night shifts and a somewhat lower level during morning shifts [23], later summarized by Åkerstedt et al. [3] Of course, the early sleep deprivation studies had shown that sleepiness (or fatigue) increased and neuropsychological performance decreased with time awake/lack of sleep [24, 25].

Objective measures of sleepiness for use in laboratory or clinical applications were pioneered by Mary Carskadon et al with their multiple sleep latency test—time to fall asleep in controlled supine position [26]. However, objective sleepiness in the individual at work was not really available. EEG measures were a natural focus as were EOG ones, but there was some skepticism of their feasibility in field studies. However, after some hesitation, we tried this approach in our first study of ambulatory EEG/EOG monitoring (Emblas again) of sleepiness in shift workers in the paper industry. We found that 20 % of the workers on the night shift showed outright sleep at work according, and subjective sleepiness was strongly increased [8]. Twenty percent also took afternoon naps after postmorning shift sleep. It should be mentioned that the participants monitored paper-mill processes in front of screens, and only occasionally left their sedate positions. Thus, the frequency of artifacts was low and the work itself somewhat soporific.

**Figure 3.**
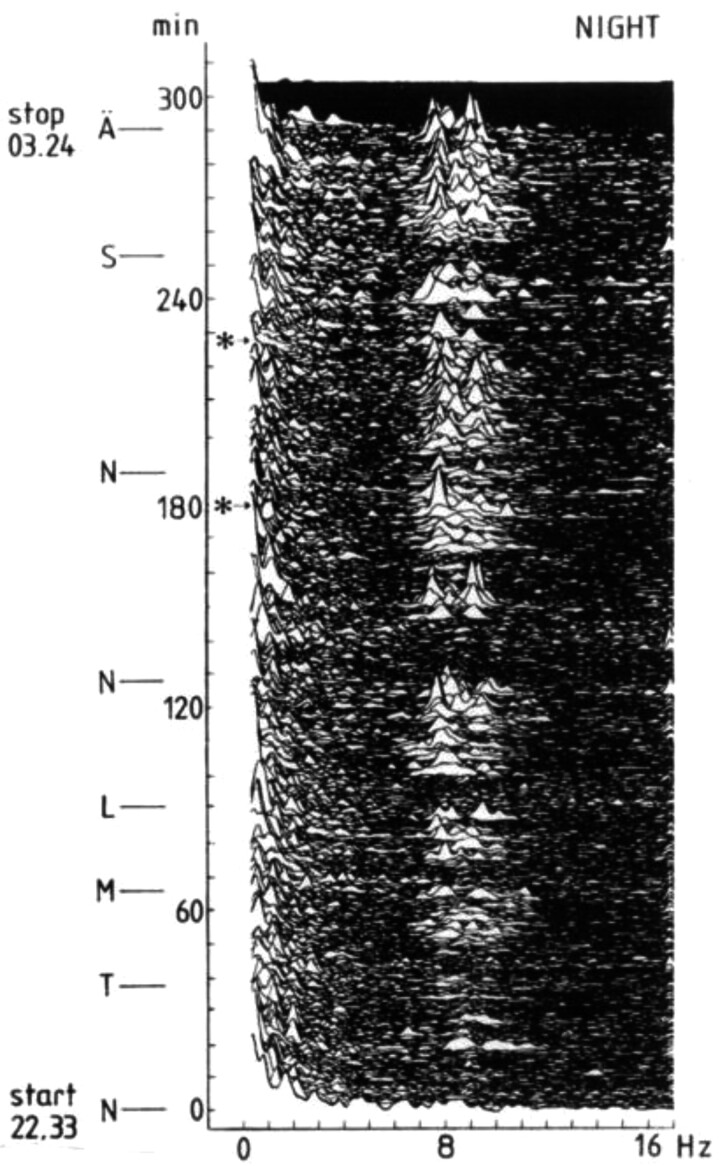
Spectral analysis of EEG of one train driver during night driving [27]. The Y-axis represents time and major stations. Asterisks indicate points of nonresponding to traffic signs. Alpha activity increases strongly after an hour’s drive. By permission from Elsevier.

In a subsequent study, we focused on train drivers, despite advice that the electromagnetic field of the engine would swamp any EEG recording. Hence, it did, but it turned out that filtering would bring it out again. In the study, we found that train drivers showed a considerable increase in EEG alpha and theta activity, during night driving [27]. On several occasions, actual falling asleep incidents (passing a red light signal without stopping, for example) occurred while driving and coincident with particularly increased alpha power density (with eyes open) and even slow eye movements (with eye lids closing) and increased subjective sleepiness (Figure 3).

In another study, twenty-five three-shift workers in a process industry were subjected to ambulatory polysomnography during one afternoon and one night shift [28]. The electroencephalographic (EEG) recordings were analyzed with spectral analysis. Subjective sleepiness increased during the night work but did not reach an extreme level. Five subjects fell asleep during night work, and the involuntary naps were preceded by a few minutes of increased alpha (8–11.9 Hz) power density. Alpha and theta activity occurred in very short bursts. The hourly mean EEG alpha power density increased significantly but moderately during the night shift and correlated with subjective ratings of sleepiness.

On the whole, it appeared that the EEG and EOG could be used to identify severe sleepiness in work situations.

## Measure of Subjective Sleepiness—the Karolinska Sleepiness Scale (KSS)

The field studies of paper mill workers and train drivers indicated that sleepiness could be measured objectively during important safety sensitive work situations. This led us to take a closer look at objective and subjective sleep in the laboratory and to develop a simple way of measuring subjective sleepiness directly linked to objective sleepiness. In a first study, we simply wanted to establish a link between EEG and EOG changes and performance at peak sleepiness—defined as 0400 h without prior sleep. We used a simple visual attention test and linked lapses to EEG/EOG predictors. The results were quite clear-cut, misses were linked to slow rolling moments (with closing eyes), and strongly increased alpha activity, but, to a smaller degree, also to theta activity [29].

The first experiment was followed up by a similar one, where we followed individuals at 2-hour intervals while moving freely in the laboratory or sitting with eyes closed or open, looking at a spot on a screen [30]. The latter was actually an extremely simple performance task—any deviation (as judged by the EOG) from a steady focus on the spot was a lapse. EEG and EMG were recorded continuously, and sleepiness was rated on the prototype Karolinska Sleepiness Scale (KSS). The latter was structured into 9 steps. The key idea behind the scale was to have a maximum representing overpowering sleepiness that everyone could recognize—an inability to keep awake, fighting sleep. At the other end, we put extreme or normal alertness, a midpoint with intermediate sleepiness without any difficulties keeping awake. The main idea was to have an easily perceived and identified states. This intention differs from the more diffuse endpoints used by many other scales. The resulting scale was labeled: 1 = extremely alert, 3 = alert, 5 = neither alert nor sleepy, 7 = the first signs of sleepiness, and 9 = extreme sleepiness, fighting sleep, unable to keep awake. In addition, the steps in between were used but were not labeled in the first version. In a later version of the scale was added labels for intermediate steps: 2 = very alert, 4 = rather alet, 6 = some signs of sleepiness, and 8 = sleepy, some effort keeping awake. The question was if EEG/EOG changes could be reliably linked to subjective sleepiness under controlled (sitting looking at a spot) or uncontrolled (moving about in the lab) conditions. The results were clear-cut. In particular, EEG alpha activity and slow eye movements increased with closeness to maximum sleepiness around the circadian nadir. The effects were more pronounced in the controlled situation.

**Figure 4.**
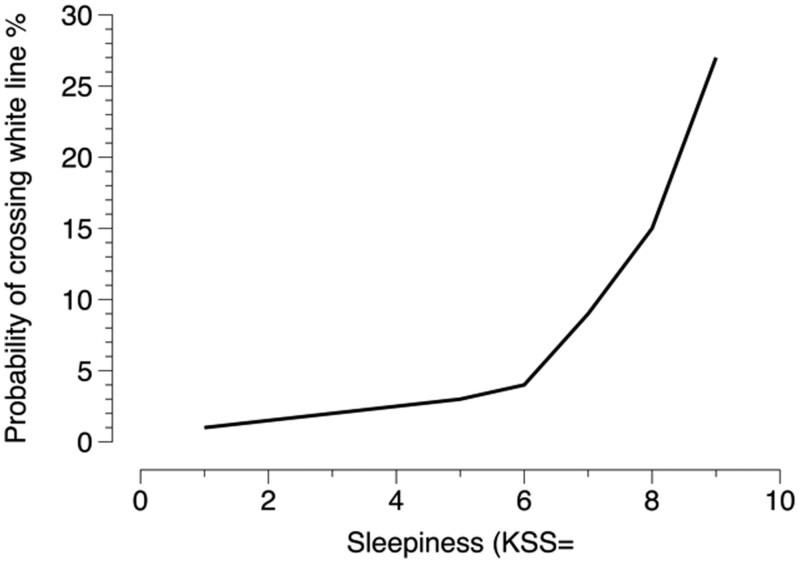
KSS and the probability of crossing a white line within 5 minutes on a country road during real driving [33].

The KSS has been used in a large number of studies and was most recently reviewed in Akerstedt et al. [31]. It has been validated against different performance measures with good success, but of particular importance is that KSS 9 is associated with being stopped from driving on a motorway within 5 minutes (by an experienced driving instructor) [32], or with a 25% risk of crossing lane markers (Figure 4) within 5 minutes on a rural road [33]. An interesting aspect on the real road tests was that local police only allowed us to drive until 0500 h in the morning and required an experienced driving inspector in the right front seat (who could stop the car in case of danger). The back seat was occupied by the experimenters. Thus, we have the feeling that the results probably underestimate the degree of sleepiness compared to the sleepiness in the average driver on the road at the same time (and in the original discussion, the local police wanted to escort the experimental vehicle each time, but yielded after discussions). The principal investigators of the studies were also pilot subjects, and all experienced the imperative demand of the eyes to close around 0300–0500 h the morning and the immense effort it took to keep them open. A very convincing experience of the power of sleepiness. It should be pointed out that not all sleepiness in this type of studies is due to night driving and sleep loss/time awake; we also found that time driven added a unit or two of sleepiness in real long-distance driving on French motorways (while controlling of time of night and time awake) [34].

In an attempt to arrive at a more narrow indicator of sleepiness risk, we used a study of post-night shift morning driving in a high fidelity, moving base car simulator. The first crash occurred at KSS = 8.1 [35]. Incidentally, highly realistic rumble strips reduced sleepiness for 5 minutes. It should be mentioned that the sleepy driving studies were run by my friend and colleague Anna Anund and her team at the Swedish Road and Transport Research Institute. We just piggy-backed on their work.

It our own interpretation of KSS levels, we usually see KSS > 7 as dangerous and 5 and 6 like a feeling of “mushiness” (typical of morning shifts and clinical burnout). Values of 3 or 4 are seen as normal daytime values, while values of 1 or 2 seldom occur unless in extreme cases. Incidentally, a day off reduces sleepiness by 1–2 units, a day of absence increases sleepiness by about 2 units [31].

One may wonder where the basis of sleepiness resides, and we often connected it with eye symptoms. This was empirically established by Filtness et al, essentially “heavy eye-lids” [36]. It should be mentioned that stimulation will temporarily increase alertness, meaning that the context of the measurement situation needs to be taken into account. Another point of some interest is that in experiments with endotoxins (lipopolysaccharides), the KSS correlates well with changes in cytokine levels [37], reflecting the increased sleepiness and fatigue involved in the sickness response.

## Model of Alertness Regulation

The two-process model of Borbély et al mentioned above dominated much of our own thinking. Simon Folkard (then in Brighton) first brought up the idea of a model of our own, based on our own data and with sleepiness being predicted (as well as sleep duration). We added a sleep inertia factor and called it the “Three process model of alertness regulation.” Later on, it was labeled the Sleep Wake Predictor (SWP) [38, 39]. Furthermore, we based our model on subjective sleepiness data from our sleep deprivation and free-run studies. The main idea of the SWP was to predict critical fatigue in relation to work scheduling or other irregularities of the sleep/wake pattern.

**Figure 5.**
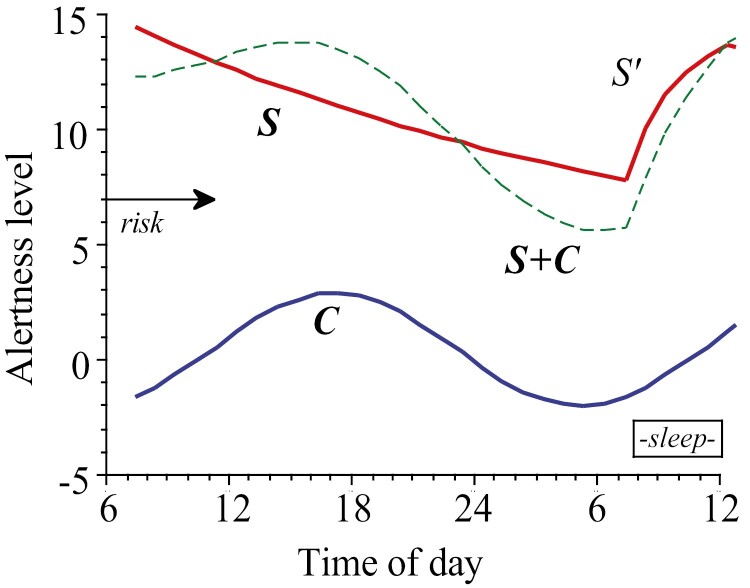
Components of the Sleep/Wake Predictor (SWP). A circadian component that is added to a homeostatic component (“S” during wakefulness and “S’” during sleep) to yield S + C = Alertness level. Sleep is terminated when level 15 is reached. Alertness level was later expressed as KSS.

The SWP has been used in accident investigations, and at present, it is the heart of the BAM (Boeing Alertness Monitor), used to advice airlines on sleepiness effects of aircrew rosters. Other models appeared, and there were actually model prediction contests during which modelers met and fought about which had the highest precision in predicting empirical data [40]. These were quite lively and stimulating meetings. I am not sure of who won, though. Possibly, we all think we did. The basic components of the SWP are illustrated in Figure 5.

## Objective Sleepiness Again

In subsequent studies of objective sleepiness during regular work, we gradually came to realize that it was not that easy to measure sleepiness objectively in real life situations. Certainly, EEG alpha and theta activity and slow eye movements were correlated with perceived sleepiness and performance degradation in our own studies (on driving) and in that of others, but we consistently found that subjective sleepiness was by far the most sensitive indicator of performance degradation, particularly in real or simulated driving (see review [31]). Hence, as formulated by Jim Horne, “people know when they are sleepy” [41, 42]. This position of Jim Horne’s was actually rather hotly contested by some colleagues, but today, subjective sleepiness is a rather well-established scientific measure. In any case, there is still a great need for a valid objective indicator of sleepiness during work or other activity.

## New Look at Shift Work

Shift work that includes night shifts was early seen as a health and safety problem [43]. However, after a number of studies of shift workers, we had the feeling that our concern with health was not taken seriously by those having such work hours. In many feedback sessions, we got the feeling that the health aspects was not that much of a concern among shift workers. They seemed to feel that their work hours were OK, also paid better than day work, and a involved a few hours shorter working time.

The findings above led us investigate what aspects of shift work that constituted a real problem for the shift workers’ point of view. I do not think this topic had really been investigated before. To eliminate confounding by local factors, we used a random national sample of shift workers. It turned out that even if three-shift work received higher problem ratings than day work, the major problems were not night or morning shifts, but rather too short head time and too short time off between shifts, as well as “social difficulties” rather than fatigue, or poor sleep, or health [44, 45]. Permanent night work was even as well liked as permanent day work (here we suspected a selection effect, but still). We concluded that we may have been barking up the wrong tree all these years. Rather embarrassing. This does not mean that the health effects should be disregarded, but rather that the problems of shift work cover a much broader spectrum than just health.

## Age, Gender, and Sleep

As expected, sleep is not the same for all individuals. Many things influence both PSG variables and self-ratings—not necessarily in the same way. Questionnaire studies indicate increasing sleep problems with increasing age and higher levels in women, as compared to men [46]. For PSG variables, the few studies available show an opposite pattern. The largest study in this area is that of Bixler et al [47], using control subjects from their sleep laboratory. They found that women had less SWS and more Stage 1 sleep than men and that this difference seemed to increase with age.

We followed up on the Bixler et al study by also introducing partial sleep deprivation (3 h of sleep during the late (0400–0700 h TIB) night on the next night PSG response in older and younger men and women [48]. As in the previous studies, women showed lower amounts of awakenings, time awake, stage N1%, higher sleep efficiency, and N3%. However, the difference was greatly enhanced in the older group, and the response (change from full night sleep to PSD) was much stronger in older women, compared to that in older men. Older men seemed to almost completely lack an N3 response to the partial sleep loss. We have recently carried out a study of > 600 men and women and confirmed the findings of poor objective sleep, and good subjective sleep, in men (Akerstedt et al, in prep). We also found that the feeling of being recovered from sleep (that is, not merely sleep quality) was higher in men. That feeling was also strongly increased in older individuals (despite a somewhat lower sleep quality).

In addition, older individuals show less negative effects on performance and emotion, than younger ones, and the resilience to sleep loss seems stronger in older individuals [49, 50].

Taken together, it seems that sleep and sleepiness are modified by age, and sex in a rather complicated pattern that needs further research.

## Stress and Other Causes of Disturbed Sleep

A number of studies (ours, and that of others) kept showing that there was a strong connection between stress and poor sleep in cross-sectional questionnaire studies [51]. Stress has also been widely blamed as a major cause of sleep disturbances [52]. There was, however, a scarcity of longitudinal studies available, and we focused on obtaining more knowledge in this area. First, using subjective ratings, we found that “hectic” work predicted later complaints of disturbed sleep [53], as did changes in work demands and work preoccupation [54]. For a review of similar research, please see Linton et al [55].

In several PSG-based longitudinal studies of real life stress and sleep, we found that stress/worries coupled to same day sleep recordings predicted modestly impaired sleep continuity [56, 57], as did teachers end-of-term stress and machine officers sleeping while on call [58, 59]. We also investigated patients with exhaustion syndrome (ICD code F43.8A in Sweden), often labeled “burnout” elsewhere. In the Swedish diagnostic rules, a main cause of the disease is exposure to long-term stress. The results showed high levels of sleep fragmentation and loss of SWS at the first recording [60]. After recovery, a year later, sleep fragmentation was down to normal levels (compared to healthy controls), and the reduction was correlated with the reduction of fatigue during the same period (SWS did not change).

The impression of our research on everyday stress and sleep is one of disappointment. There are slight impairments in PSG recorded sleep in connection with everyday stress, but it would be difficult to argue that they are of any clinical significance. The reason probably is that high stress is often difficult to predict, and individuals are unlikely to agree to being recorded at such times in life. The advent of unobtrusive, wearable, recording equipment may lead to a much needed increase in knowledge in this area.

## Mortality—Sleep Duration and Sleep Quality

As a sleep researcher, one is very often asked about the optimal duration of sleep, and quite a few studies are available [61, 62]. Essentially, both short and long sleep are associated with mortality [61, 62]. The topic has caught media’s attention more than anything in current sleep research, and one gets the feeling that individuals who do not produce 7 or 8 hours of sleep are doomed. Most sleep researchers would probably agree that this is a gross exaggeration, and I felt that more research was needed. It also meant that I needed to team up with epidemiologists, which was a new and interesting experience in scientific thinking. I was, however, slightly discouraged by one reviewer’s comment on one of our first attempts: “Yet another one of those sleep and mortality studies.”

**Figure 6.**
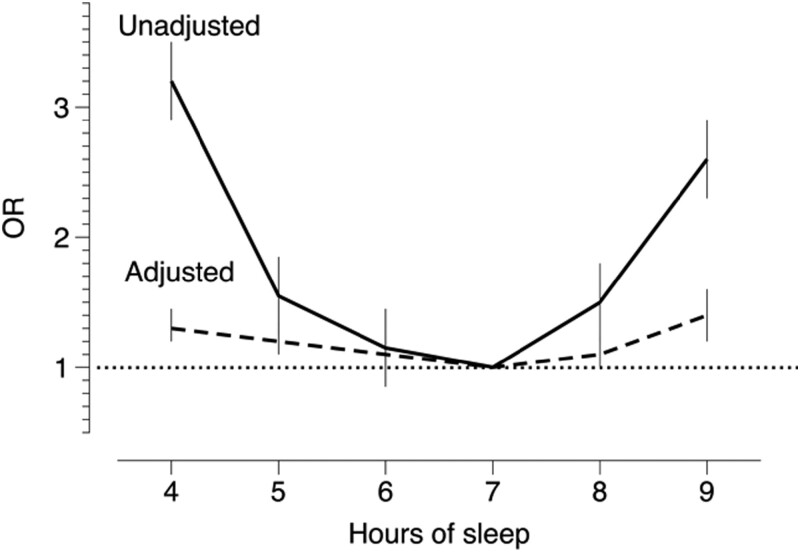
Mortality (OR, 95% confidence interval) associated with reported sleep duration. Unadjusted and adjusted for age and health. Dotted line = OH for reference value (7 h).

Our first attempt focused on age, since it was usually only adjusted for in previous work, not used as a variable. We found that there was a long span of sleep durations 5.5–8 h without any link to increased mortality [63] that the association was mainly present in older (>50 years) age groups, and even then, it was quite weak. Figure 6 illustrates the results for unadjusted data and after adjustment for age and health (health effects were minor). In terms of gender, women appeared to have a higher sensitivity to short sleep (≤5 h) in terms of mortality than men and actually rated short sleep more negatively than men [64]. In addition, those who compensated short weekday sleep with sleeping in during the weekend did not have an increased risk [65]. A rather popular finding it turned out. Still, much more work needs to be done, especially on the influence of prebaseline latent disease (which may affect both sleep and mortality), and on the influence of age and gender, as well as the influence of disease during follow-up, and of lifestyle.

## Objective and Subjective Sleep Quality

Having frequently brought up both subjective and objective aspects of sleep, it might be appropriate to bring out the embarrassing discrepancy between them. To paraphrase Jim Horne’s statement on sleepiness, “people do not really know how good or poorly they sleep.” Thus, polysomnographical data recorded in connection with the construction of major sleep quality questionnaires showed almost no correspondence between objective and subjective sleep. This was the case with the later widespread Pittsburgh Sleep Quality Index (PSQI) [66], as well as with the very popular Insomnia Severity Index (ISI) [67]. A number of studies have addressed the objective/subjective sleep issue, usually finding a mismatch between questionnaire and PSG results. One exception is the meta-analysis of Baglioni et al which showed small, but significant [68], PSG differences between insomniac and control groups. For example, TST differed (significantly) only 21 minutes between the two groups, which is not likely to be seen as a problem.

In an early attempt to understand more of the lack of correspondence between objective and subjective sleep, we collaborated with our British colleagues James Waterhouse and David Minors in Manchester. They collected longitudinal PSG data across 2 weeks and also used ratings of different aspects of sleep quality. We found that subjective sleep quality showed considerable correlations with PSG parameters [69–71]. We also found that subjective sleep quality was associated with long TST and high SWS levels across a 18 4 h sleep episodes at different times of day. Interestingly, high levels of SWS were also associated with high sleepiness, probably related to difficulties waking up when SWS reached high levels.

The longitudinal studies were, obviously, based on intraindividual associations over time, which made us think that the subjective/objective correspondence was more easily brought out within individuals. Interindividual difference would then be controlled for or maybe it was the fact that the ratings were obtained from exactly the same sleep as was recorded polysomnographically, whereas studies with interindividual approaches compare different individuals with good or poor sleep, which is subject to different ways of interpreting the objective qualities of their sleep.

An interesting insight in the objective/subjective sleep issue was brought out by Allison Harvey’s qualitative work on the meaning of insomnia, which showed that both insomniacs and noninsomniacs characterized “poor sleep” as a feeling of fatigue or related states, and less frequently as increases in quantitative measures like sleep latency, time awake, and similar indicators [72]. This suggested that sleep problems were not clearly related to “not sleeping,” as has been the usual assumption.

To understand more, we analyzed PSG data in 350 women (men had not been recorded) together with our new friends at Uppsala University and found that subjective sleep quality indices correlated rather well with PSG variables for a particular sleep. This was the case, especially for sleep continuity variables like sleep efficiency, number of awakenings, early morning awakenings, etc. However, when we compared the PSG variables with questionnaire ratings of “habitual” sleep, for example, “how often do you have difficulties waking up from sleep without being able to return to sleep?” [73], we found no associations. Instead, rated fatigue and C-reactive protein (CRP) were associated with ratings of “habitual” sleep quality. This suggested that something in the retrospective generalization of sleep quality made it differ from a particular rated sleep. It could, of course, be that one single sleep would not be representative of a number of sleeps across several months. It could be that long-term estimates of sleep quality are based on perception of other factors not directly relevant to sleep [73].

In the study cited, we also found that older individuals rated higher sleep quality than younger ones for the same PSG value. It, thus, seemed as if tolerance for objectively poor sleep increases with age. This is interesting in its own right but would also reduce the association between objective and subjective sleep in a sample with a broad age span.

Presently, we are analyzing data from large study of both men and women, comparing their ratings of habitual sleep, with ratings of a specific recorded sleep, as well as with its PSG. We hope this will shed more light on the subjective/objective sleep issue.

## Final Word

Sleep has been, and is, a fascinating area of research. In the beginning, it may have been because so little was known, it was easy to contribute, and there was a pioneer spirit. Now, it may be because sleep has begun to address life and death matters, and there is a sense that important breakthroughs wait around the corner. An interesting time for new (and old) sleep researchers!

## Notes

This paper is part of the Living Legends in Sleep Research series, which is sponsored by Idorsia Pharmaceuticals and Jazz Pharmaceuticals.
